# Supplementary material for: 3D genome organization contributes to genome instability at fragile sites
Source: Nat Commun. 2020 Jul 17;11:3613. doi: 10.1038/s41467-020-17448-2 (PMC7367836; doi:10.1038/s41467-020-17448-2)
Supplement: Supplementary file 1 — Supplementary Information [file 41467_2020_17448_MOESM1_ESM.pdf]

Supplementary Figure 1

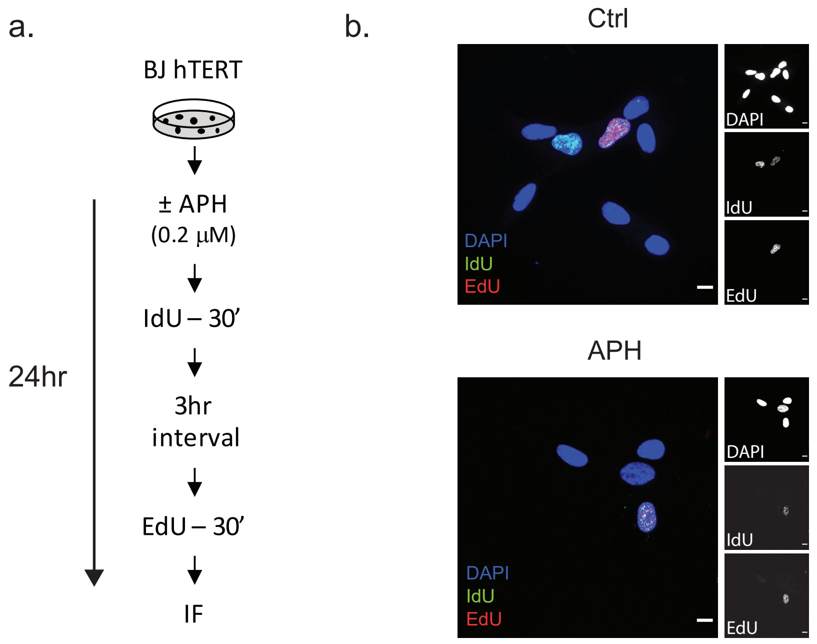

c.

	Ctrl	APH
Total nuclei	417	383
Double labeled (Still in S-phase)	70	97
First label only (Exited S-phase)	20	26
Second label only (New entry to S-phase)	7	11
No label (Non-replicating)	320	249

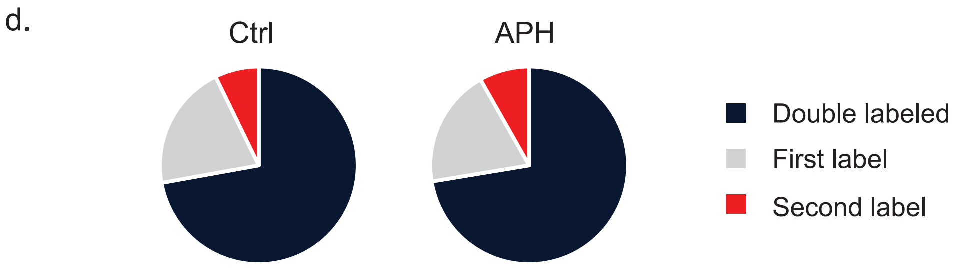

Supplementary Fig. 1. Mild replication stress does not alter S-phase progression. (**a**) Schematic description of the pulse-chase-pulse assay. BJ-hTERT cells with (+) or without (-) APH treatment for 24 hours were labeled with IdU for 30 minutes, 4 hours prior to fixation, washed and labeled with EdU for 30 minutes prior to fixation. (**b**) Representative images of BJ-hTERT cells labeled with IdU and EdU, with (APH) or without (Ctrl) APH treatment. (**c**) A table summarizing the S-phase populations of APH-treated (APH) and untreated (Ctrl) cells. (**d**) Pie charts summarizing the S-phase populations as in **c**. Scale bars: 10 μm. Source data are provided as a Source Data file.

Supplementary Figure 2

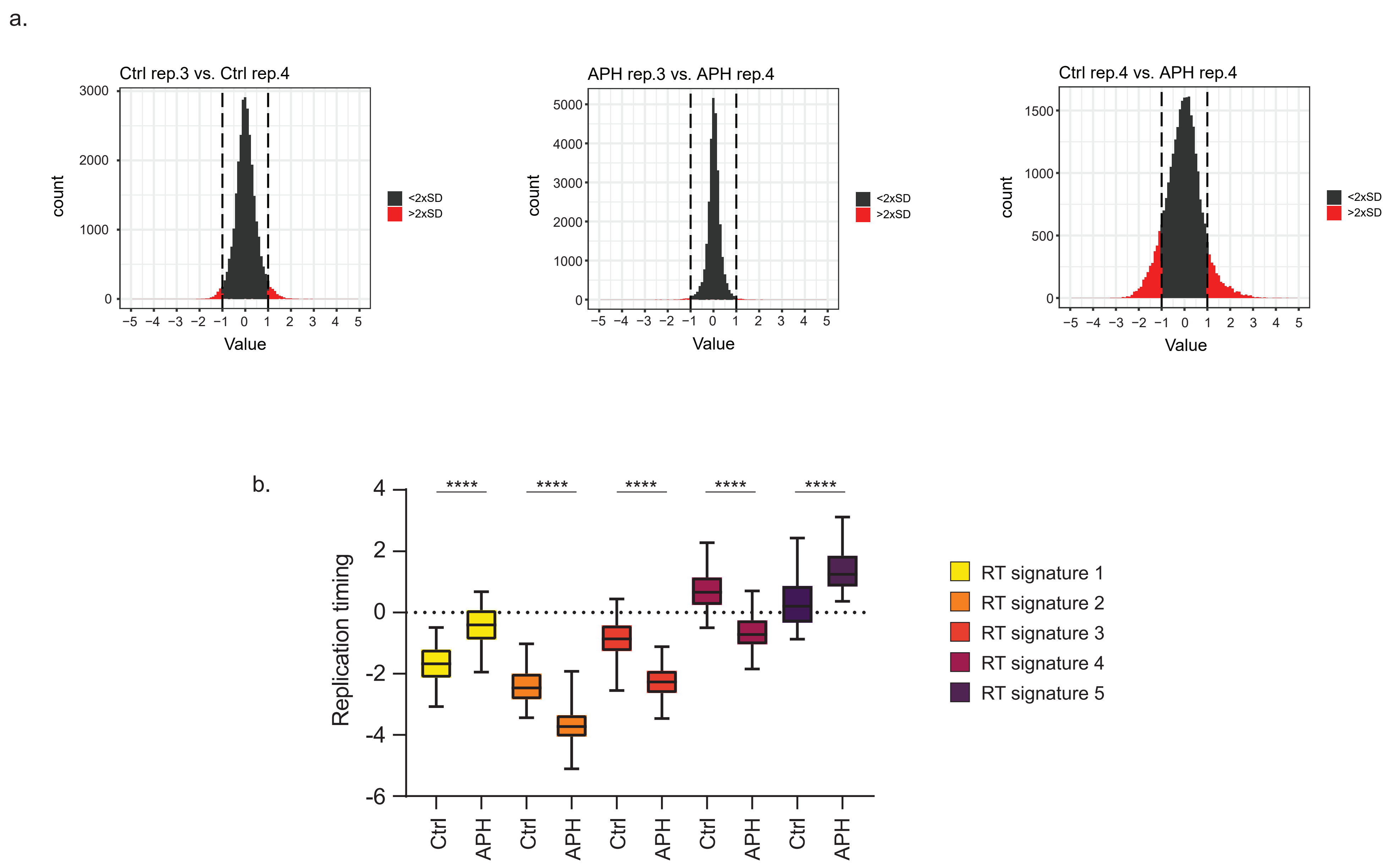

Supplementary Fig. 2. Identification of RT signatures in BJ-hTERT cells treated with APH. (**a**) RT differences between Ctrl and APH-treated cells (right panel) are larger than the differences between technical replicates within each condition (left and center panels). RT-variable regions were identified as those that display differences ≥1 (equal to two standard deviations in pairwise comparisons of technical repeats). RT of Ctrl and APH replicates 3 and 4 are presented as an example. In black are values < 1 RT unit (2xSD) and in red are values ≥1 RT unit (2xSD). (**b**) Within each RT signature, genomic regions of identified variable RT replicate significantly differently in Ctrl and APH-treated cells, based on pairwise two-sided t-test with Bonferroni adjustment. RT signatures are color coded as indicated. RT signature 1 (n = 354); RT signature 2 (n = 287); RT signature 3 (n = 252); RT signature 4 (n = 180); RT signature 5 (n = 204). N represents the number of RT 100kb windows identified as changing following APH treatment. The bounds of the box: 25th and 75th percentiles; center line: median; box range: min and max values. **** $p < 0.0001$.

Supplementary Figure 3

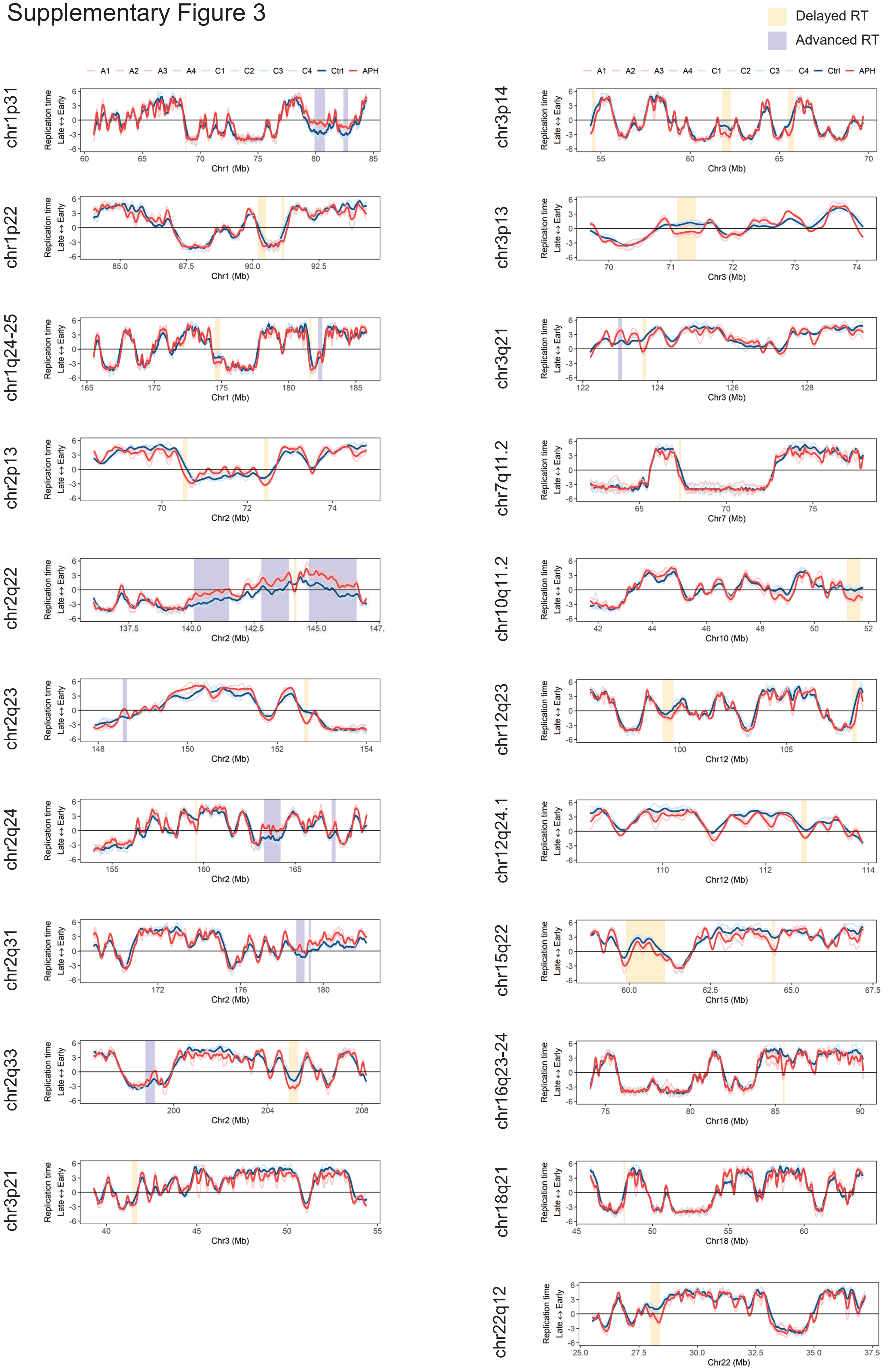

Supplementary Fig. 3. CFSs are enriched for delayed RT signatures. RT profiles of fragile cytogenetic bands showing delayed RT signatures (orange boxes) and advanced RT signatures (blue boxes) following APH treatment. Four replicates per condition are presented (1-4) color coded according to the legend (Ctrl in blue, APH in red).

Supplementary Figure 4

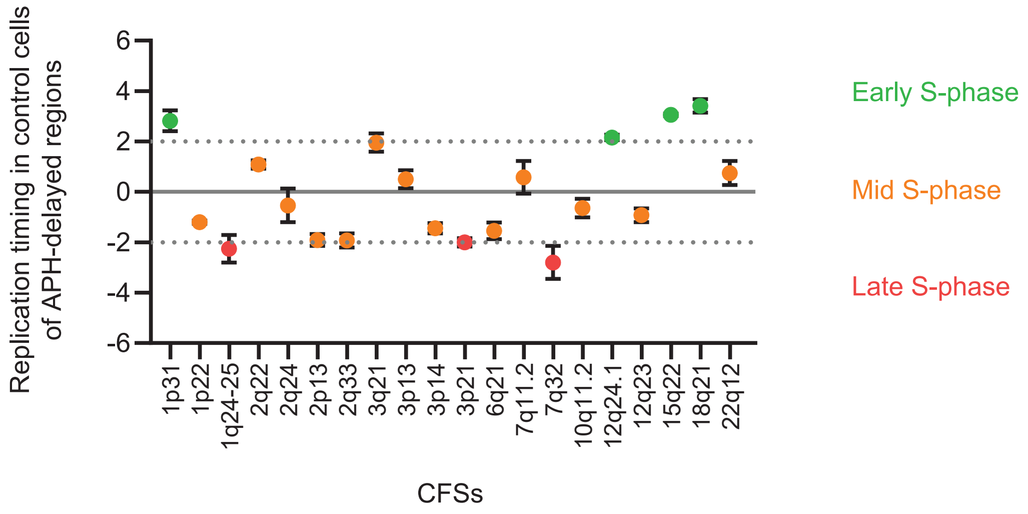

Supplementary Fig. 4. Delayed RT loci at CFSs are not late-S replicating. RT of loci delayed by APH treatment, in unperturbed BJ-hTERT cells (control) showing they are not late S-phase replicating loci. Data are presented as the mean of RT profile ± SD (N=4). Each dot is color coded according to its time of replication: early-S in green; mid-S in orange; late-S in red. Source data are provided as a Source Data file.

Supplementary Figure 5

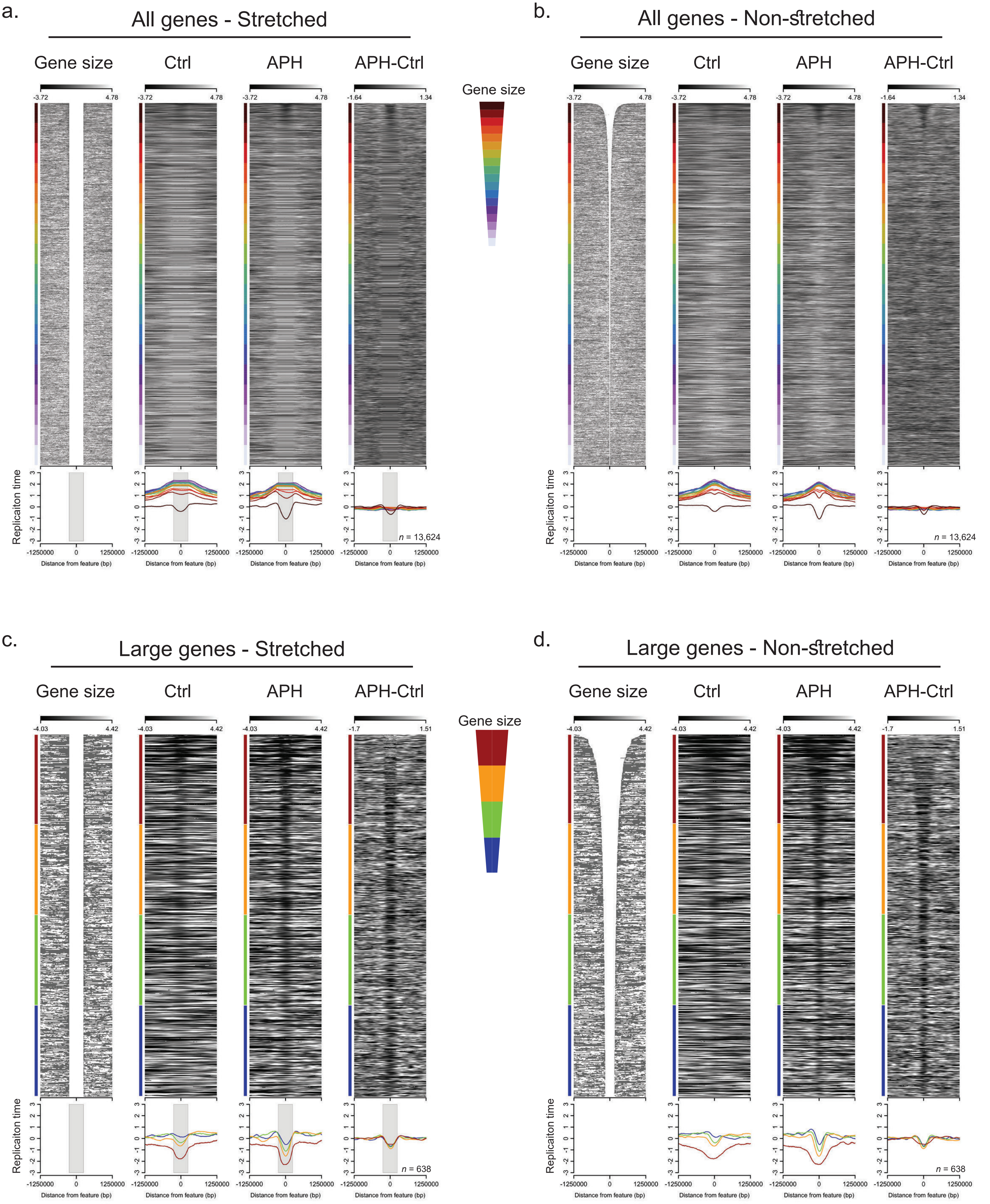

Supplementary Fig 5. Related to Fig 3. Large genes are delayed by APH. (**a,b**) Heat maps and averaged RT profiles of all genes clustered by size in Ctrl and APH-treated cells. The difference in RT following APH treatment is presented as the subtraction of the RT in Ctrl from the RT in APH-treated cells (APH-Ctrl). (**a**) Genes are centered at 0, and stretched to fit the grey box. (**b**) Genes are centered at 0 but are not stretched. One Mb flanking the gene upstream and downstream are presented. Left panel shows a heat map of gene sizes for stretched (**a**) and non-stretched (**b**). Heat maps show individual genes sorted according to size from top to bottom (largest to smallest, respectively) and color coded by size as indicated. (**c,d**) Heat maps and averaged RT profiles of large genes (>300 kb) clustered by size represented as in **a,b**.

Supplementary Figure 6

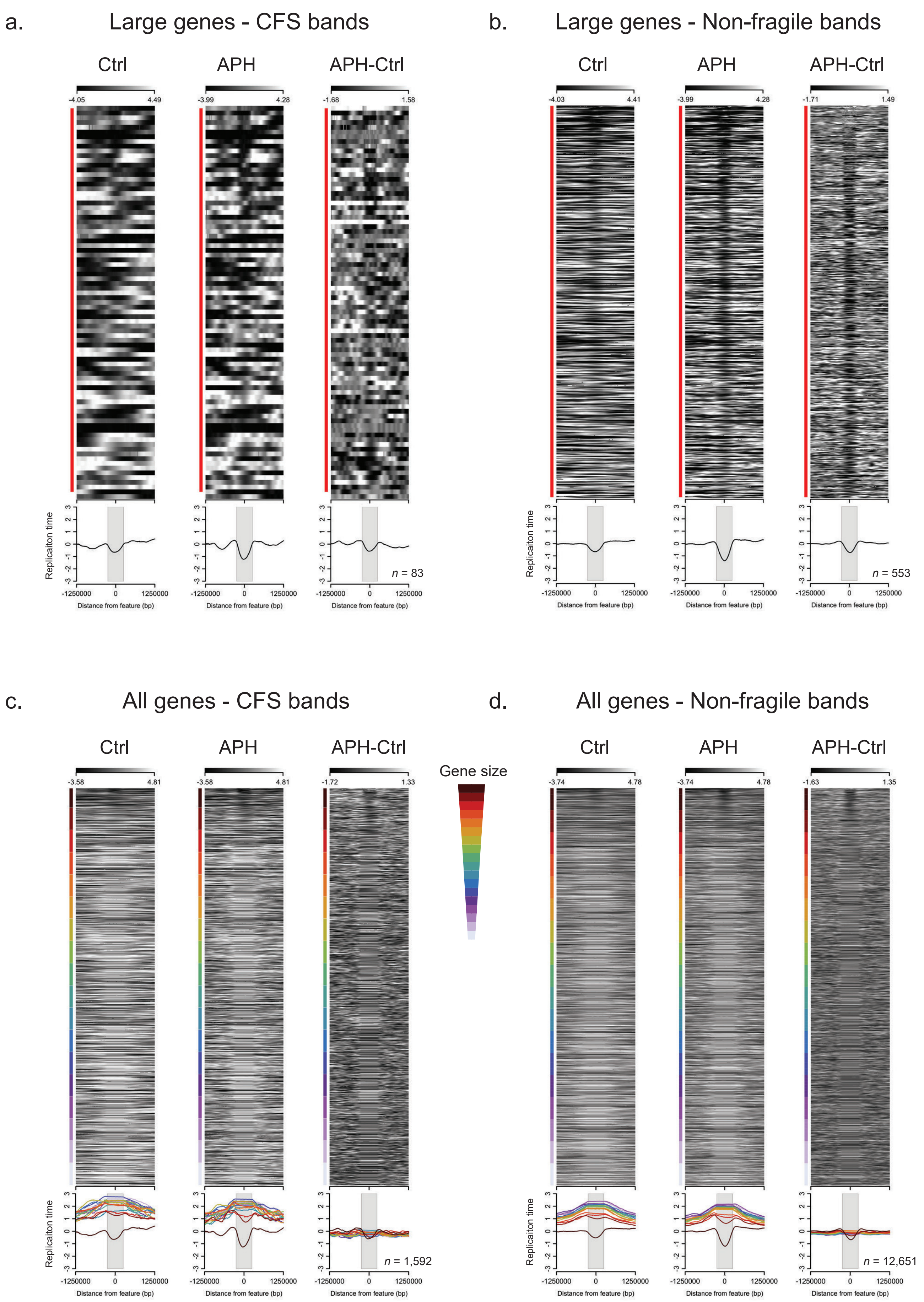

Supplementary Fig. 6. Related to Fig. 3. Large genes are delayed by APH irrespective to fragile site bands. Heat maps and averaged RT of large genes (>300 kb) within CFS bands (**a**) and in non-fragile bands (**b**) in Ctrl and APH-treated cells. The difference in RT following APH treatment is presented as the subtraction of the RT in Ctrl from the RT in APH-treated cells (APH-Ctrl). Genes are centered at 0 and stretched to fit the grey box, and 1 Mb upstream and downstream the genes are presented. Heat map shows individual genes, which are sorted according to size from top to bottom (largest to smallest, respectively). (**c,d**) Heat maps and averaged RT of all genes within CFS bands (**c**) and in non-fragile bands (**d**), are represented as in **a,b**. Heat maps show individual genes sorted according to size from top to bottom (largest to smallest, respectively) and color coded by size as indicated.

Supplementary Figure 7

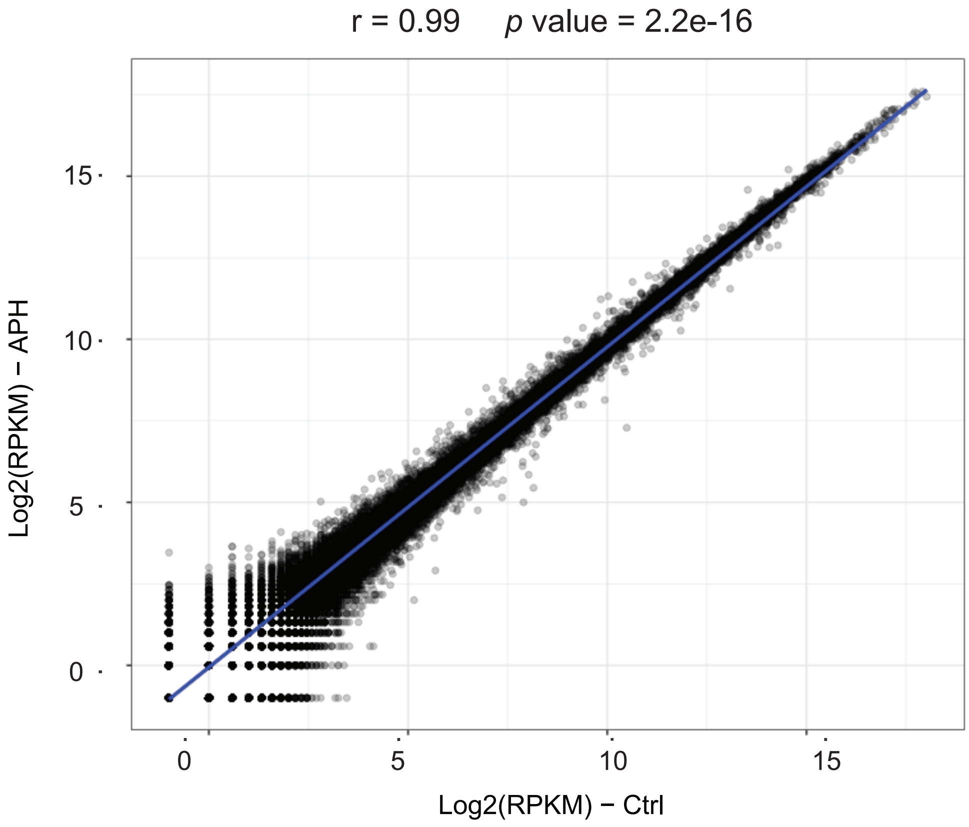

Supplementary Fig. 7. APH treatment does not affect transcription. Scatter plot of mean expression values [Log2(RPKM)] of all genes in APH-treated (APH) and untreated (Ctrl) BJ-hTERT cells. Each dot is the mean expression value of N = 2. No gene was found to be differentially expressed using EdgeR (please see Methods). Pearson's correlation coefficient = 0.99, *p* value = 2.2e-16.

Supplementary Figure 8

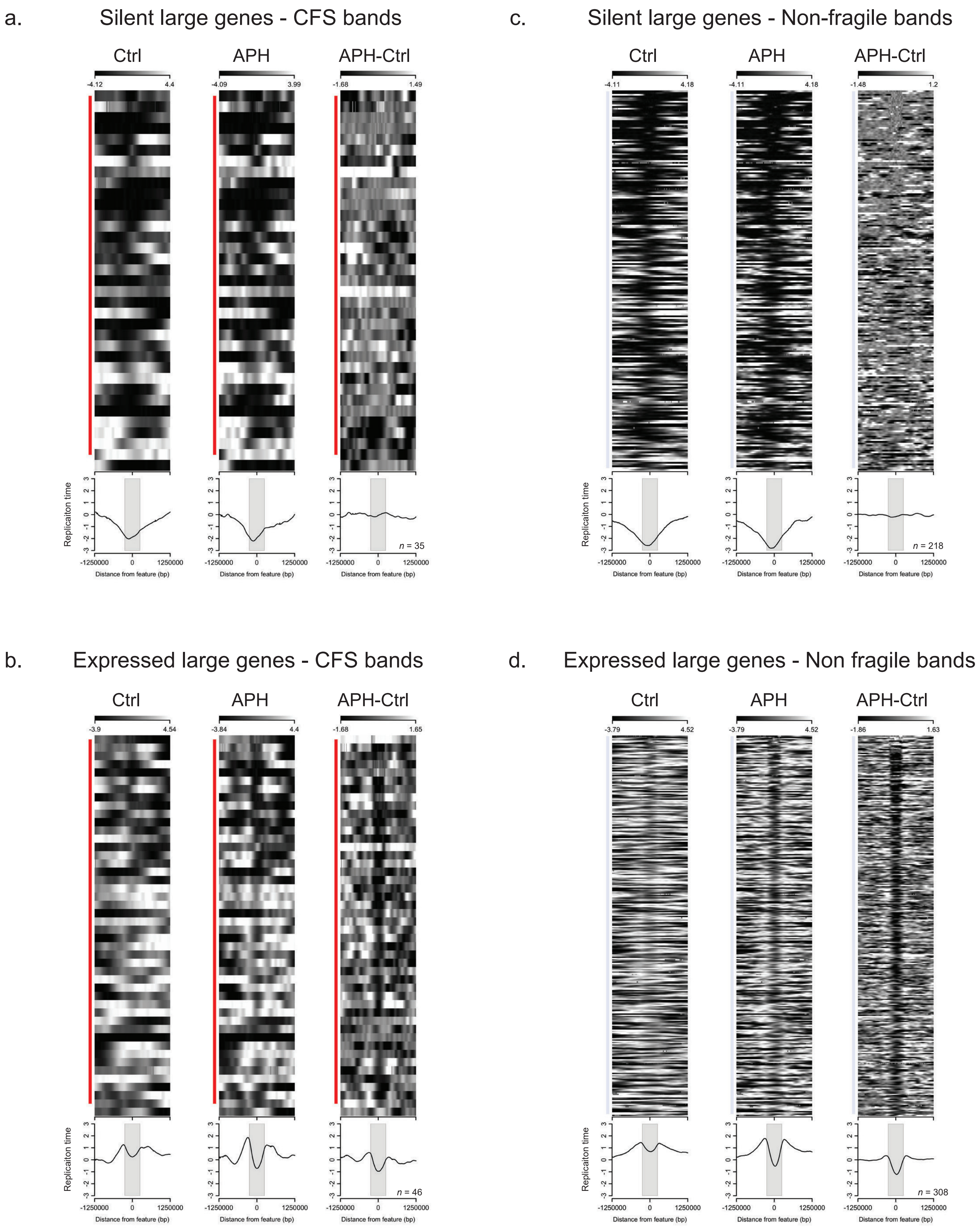

Supplementary Fig. 8. Related to Fig. 3. Expressed large genes are delayed by APH irrespective to fragile site bands. Heat maps and averaged RT of silent (**a**) and expressed (**b**) large genes (>300 kb) within CFS bands in Ctrl and APH-treated cells. The difference in RT following APH treatment is presented as the subtraction of the RT in Ctrl from the RT in APH-treated cells (APH-Ctrl). Genes are centered at 0 and stretched to fit the grey box, and 1 Mb upstream and downstream the genes are presented. Heat map shows individual genes, which are sorted according to size from top to bottom (largest to smallest, respectively). (**c,d**) Heat maps and averaged RT of silent (**c**) and expressed (**d**) large genes located in non-fragile bands, are represented as in **a,b**.

Supplementary Figure 9

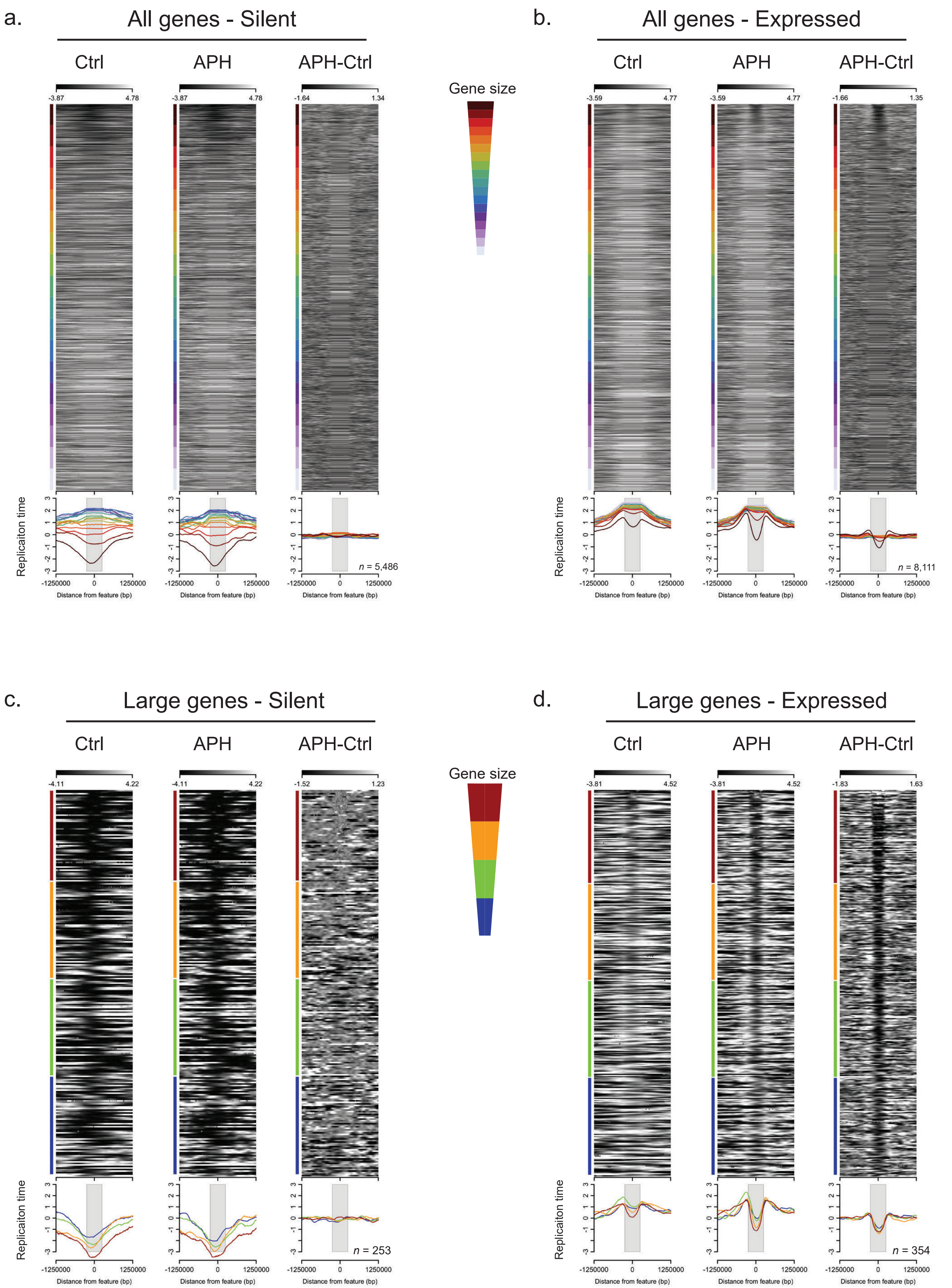

Supplementary Figure 9. Expressed large genes are delayed by APH. Heat maps and averaged RT of all genes transcriptionally silent (**a**) and transcriptionally active, expressed (**b**) in Ctrl and APH-treated cells. The difference in RT following APH treatment is presented as the subtraction of the RT in Ctrl from the RT in APH-treated cells (APH-Ctrl). Genes are centered at 0 and stretched to fit the grey box, and 1 Mb upstream and downstream the genes are presented. Heat map shows individual genes, which are sorted according to size from top to bottom (largest to smallest, respectively) and color coded by size as indicated. (**c,d**) Heat maps and averaged RT of large genes (>300 kb) transcriptionally silent (**c**) and transcriptionally active, expressed (**d**) are represented as in **a,b**.

Supplementary Figure 10

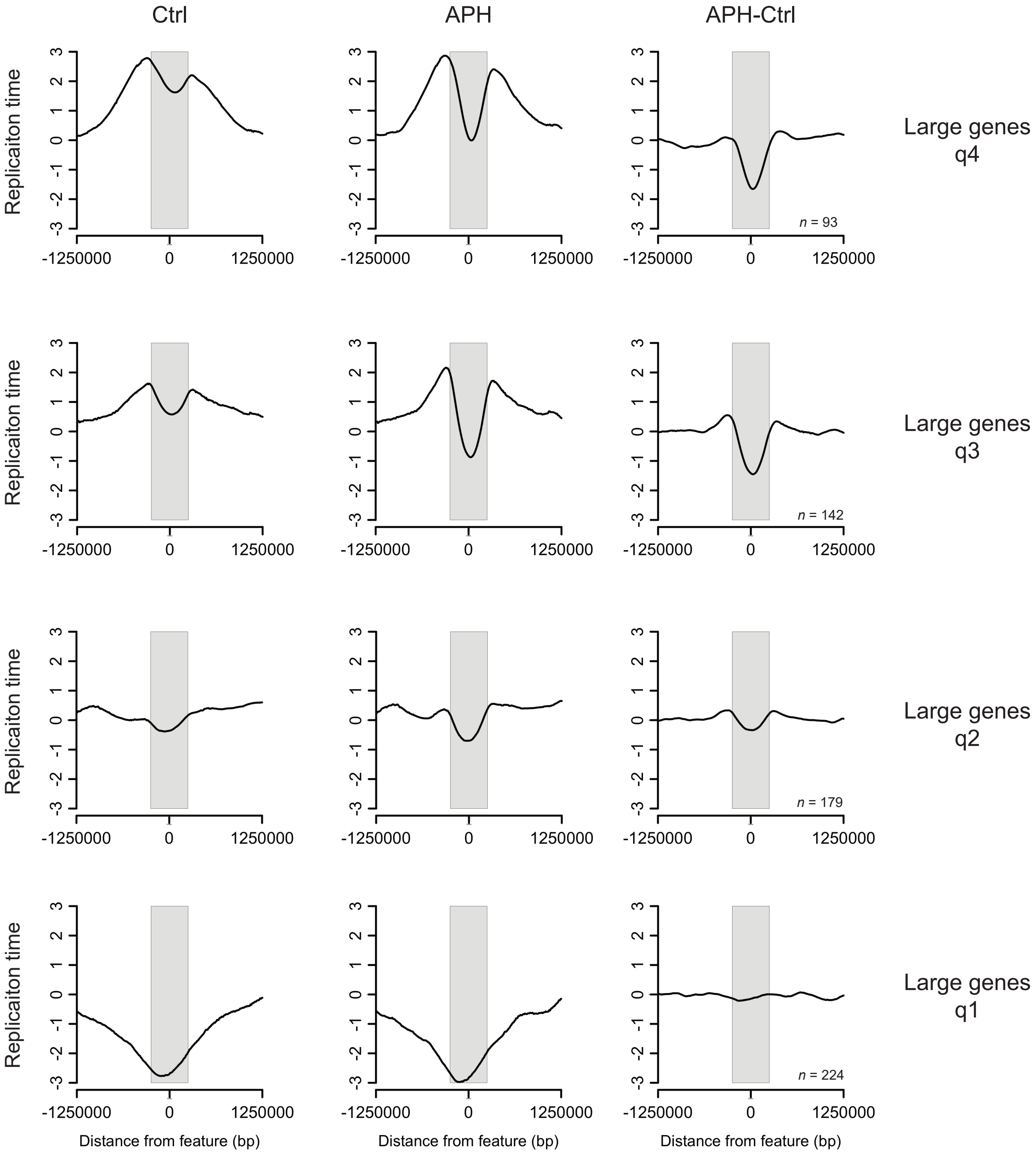

Supplementary Fig. 10. Large genes expressed above the median level are delayed by APH. Averaged RT of large genes (>300 kb) binned by whole-genome expression quartile 1-4, where q1 is the lowest expression and q4 is the highest expression level are presented in Ctrl and APH-treated cells. The difference in RT following APH treatment is presented as the subtraction of the RT in Ctrl from the RT in APH-treated cells (APH-Ctrl). Genes are centered at 0 and stretched to fit the grey box, and 1 Mb upstream and downstream the genes are presented.

Supplementary Figure 11

a.

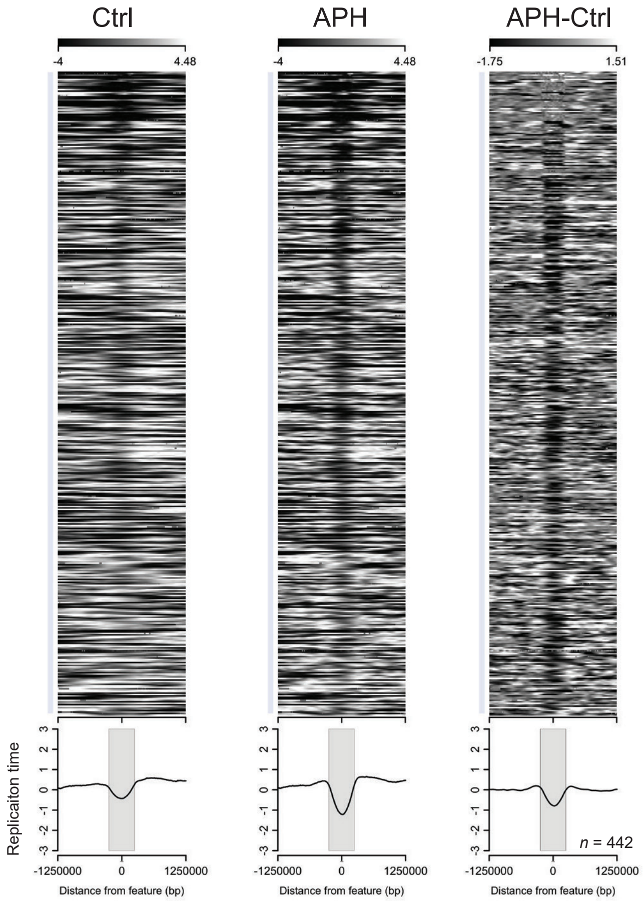

Large genes
Inter-TAD

b.

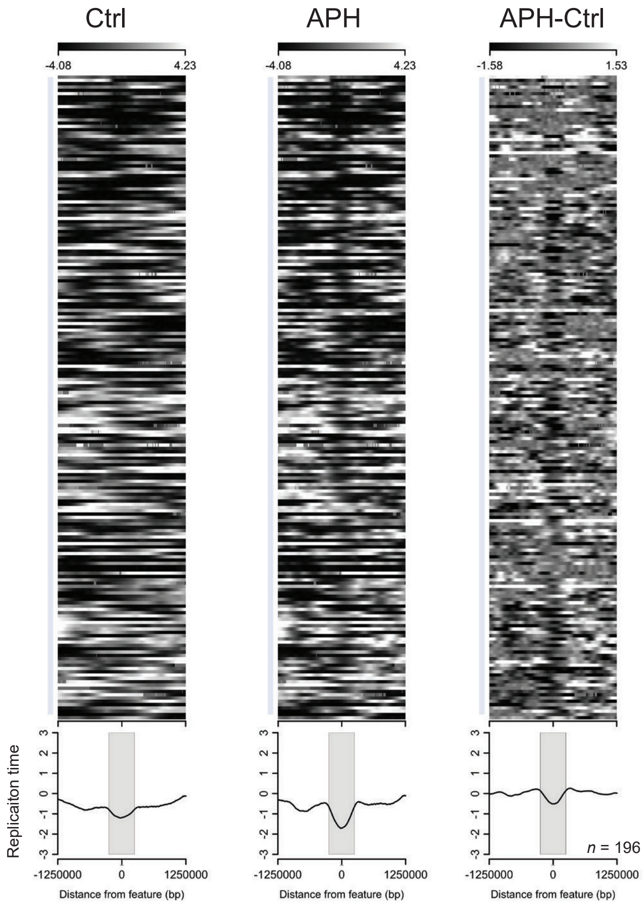

Large genes
Intra-TAD

Supplementary Fig. 11. Large genes are delayed by APH irrespective to their 3D architecture. Heat maps and averaged RT of large (>300 kb) spanning over a TAD boundary (inter-TAD) (**a**) and located within a TAD domain (intra-TAD) (**b**) in Ctrl and APH-treated cells. The difference in RT following APH treatment is presented as the subtraction of the RT in Ctrl from the RT in APH-treated cells (APH-Ctrl). Genes are centered at 0 and stretched to fit the grey box, and 1 Mb upstream and downstream the genes are presented. Heat map shows individual genes, which are sorted according to size from top to bottom (largest to smallest, respectively).

Supplementary Figure 12

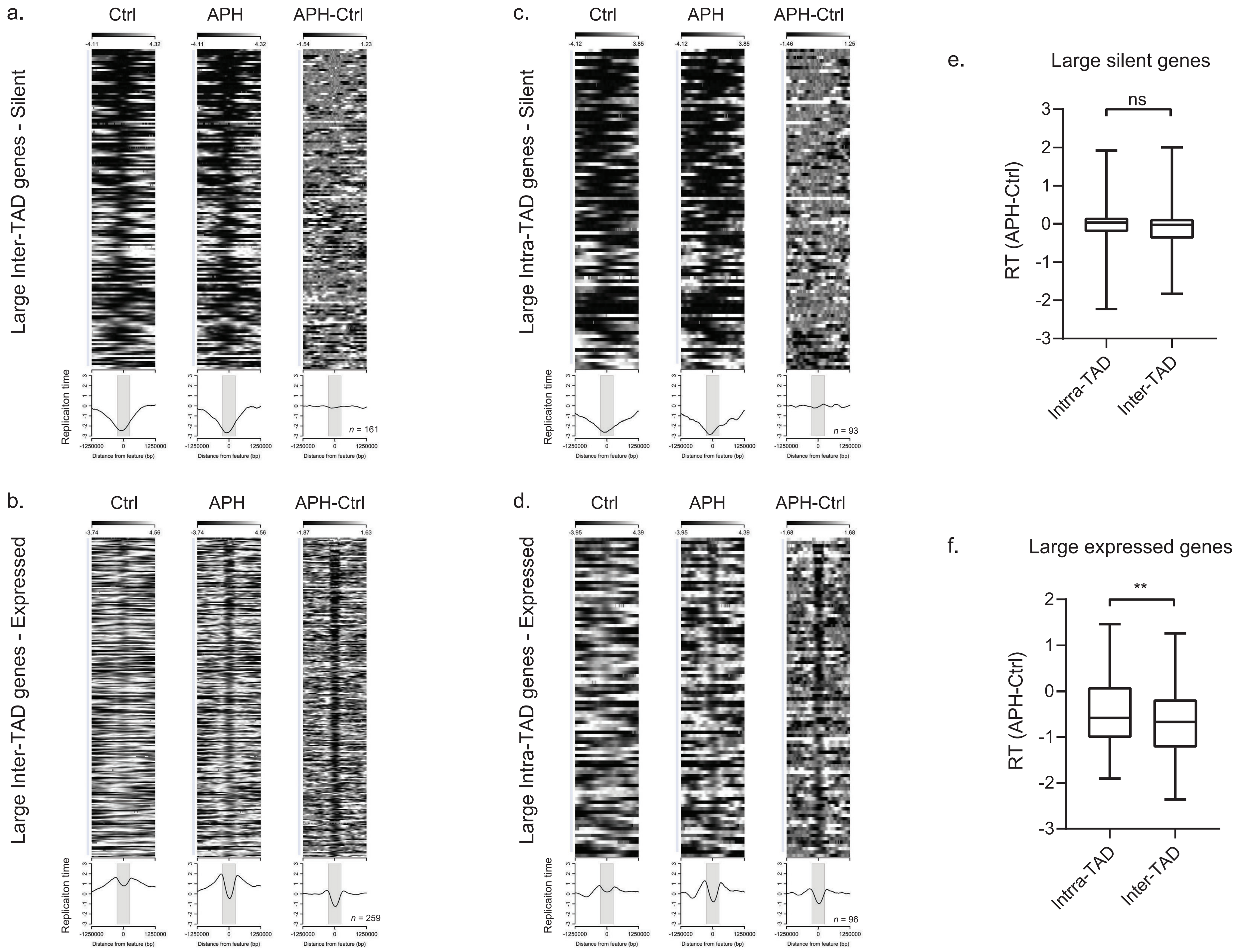

Supplementary Fig. 12. Expressed large genes spanning over a TAD boundary are delayed by APH. Heat maps and averaged RT of large genes (>300 kb) spanning over a TAD boundary (inter-TAD) transcriptionally silent (**a**) and transcriptionally active, expressed (**b**) in Ctrl and APH-treated cells. The difference in RT following APH treatment is presented as the subtraction of the RT in Ctrl from the RT in APH-treated cells (APH-Ctrl). Genes are centered at 0 and stretched to fit the grey box, and 1 Mb upstream and downstream the genes are presented. Heat map shows individual genes, which are sorted according to size from top to bottom (largest to smallest, respectively). (**c,d**) Heat maps and averaged RT of large genes (>300 kb) located within a TAD domain (intra-TAD) transcriptionally silent (**c**) and transcriptionally active, expressed (**d**) are represented as in **a,b**. (**e**) Boxplot of the difference in averaged RT (APH-Ctrl) of silent large genes located within a TAD domain (intra-TAD, n = 93) and overlapping a TAD boundary (inter-TAD, n = 161). (**f**) Boxplot of the difference in averaged RT (APH-Ctrl) of expressed large genes located within a TAD domain (intra-TAD, n = 96) and overlapping a TAD boundary (inter-TAD, n = 259). N represents the number of genes analyzed. The bounds of the box: 25th and 75th percentiles; center line: median; box range: min and max values. Two sided Mann-Whitney rank-sum test, ns – Non significant (P = 0.2437); ** $p < 0.01$ (P = 0.0092).

Supplementary Figure 13

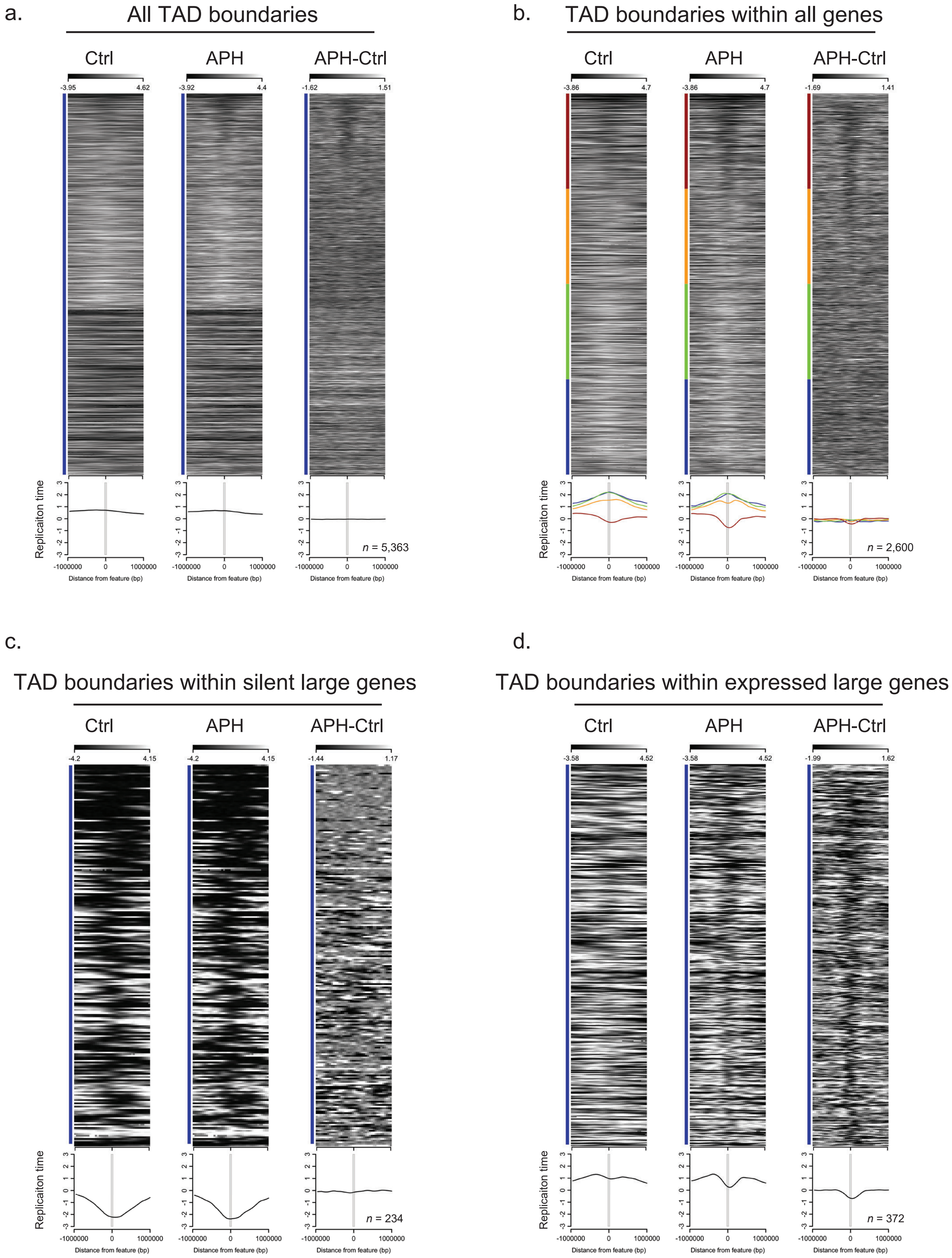

Supplementary Fig. 13. TAD boundaries are located at the core RT-delay of expressed large genes. (**a**) Heat maps and averaged RT of all TAD boundaries in Ctrl and APH-treated cells. The difference in RT following APH treatment is presented as the subtraction of the RT in Ctrl from the RT in APH treated cells (APH-Ctrl). TAD boundaries are centered at 0 and stretched to fit the grey box, and 800 kb upstream and downstream the TAD boundaries are presented. Heat map shows individual TAD boundaries, which are sorted according to size of genes they are located within from top to bottom (largest to smallest to TAD boundaries non-overlapping any gene). (**b**) Heat maps and averaged RT of TAD boundaries located within all genes are represented as in **a**. Heat map shows individual TAD boundaries, which are sorted according to size of genes they are located within from top to bottom (largest to smallest, respectively) and color coded as indicated. (**c,d**) Heat maps and averaged RT of TAD boundaries located within large genes (> 300 kb) transcriptionally silent (**c**) and transcriptionally active, expressed (**d**) are represented as in **a**.

Supplementary Figure 14

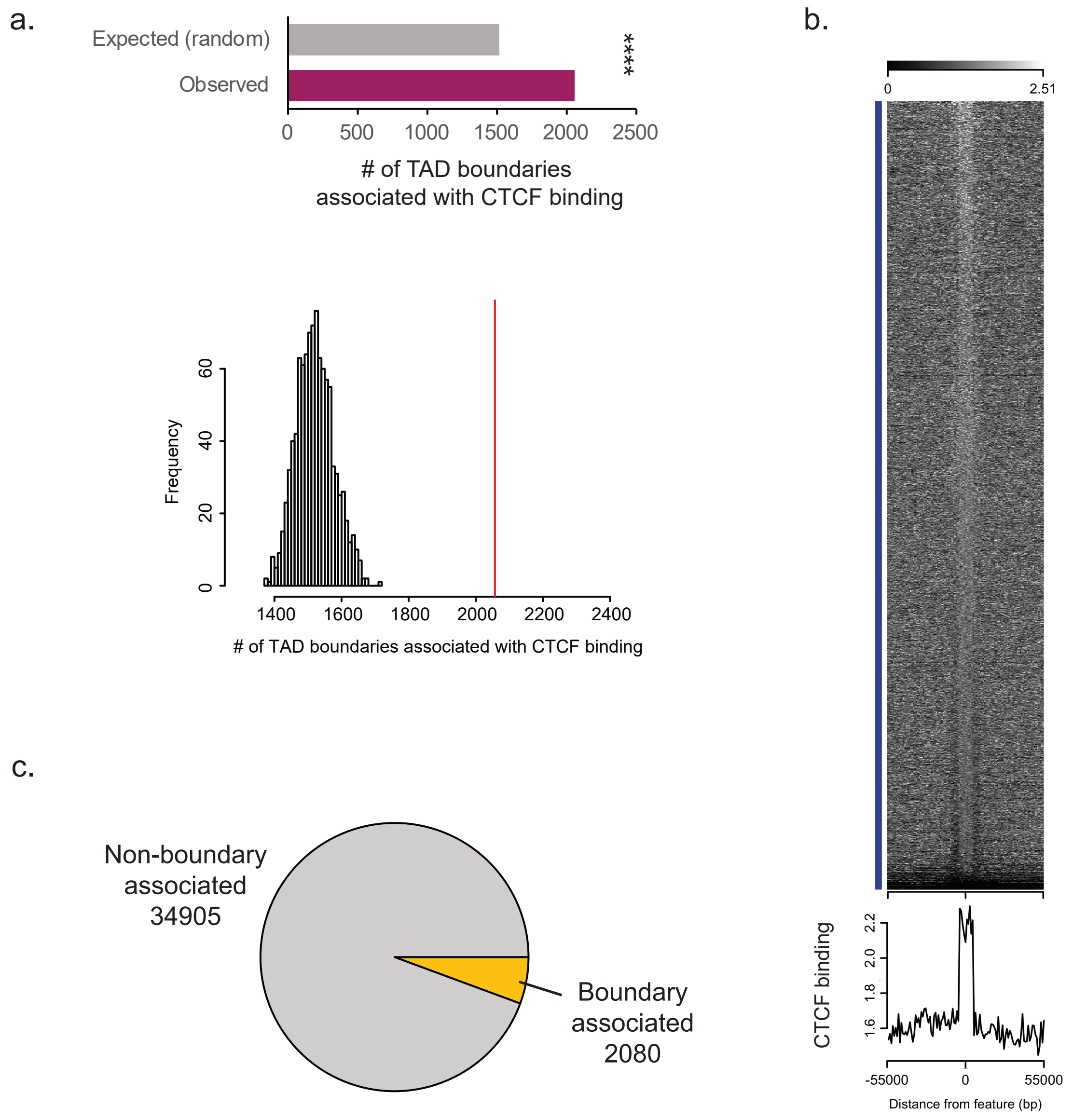

Supplementary Fig. 14. TAD boundaries are associated with CTCF binding, however, CTCF binding is genome wide. (**a**) TAD boundaries are associated with CTCF binding sites above the randomly expected level. Top: observed number of TAD boundaries with CTCF binding sites in HFF cells (magenta) and averaged number of expected boundaries by random shuffling of TAD boundaries across the genome (grey); 1,000 iterations performed. Bottom: histogram of the number of randomly selected TAD boundaries associated with CTCF. (**b**) Heat map and averaged CTCF binding at TAD boundaries. Boundaries are centered at 0 and stretched to a length of 10kb, flanking 100kb are shown. (**c**) Pie chart of CTCF binding sites associated with TAD boundaries or non-boundary regions. **** $p < 0.0001$, permutation test.

Supplementary Figure 15

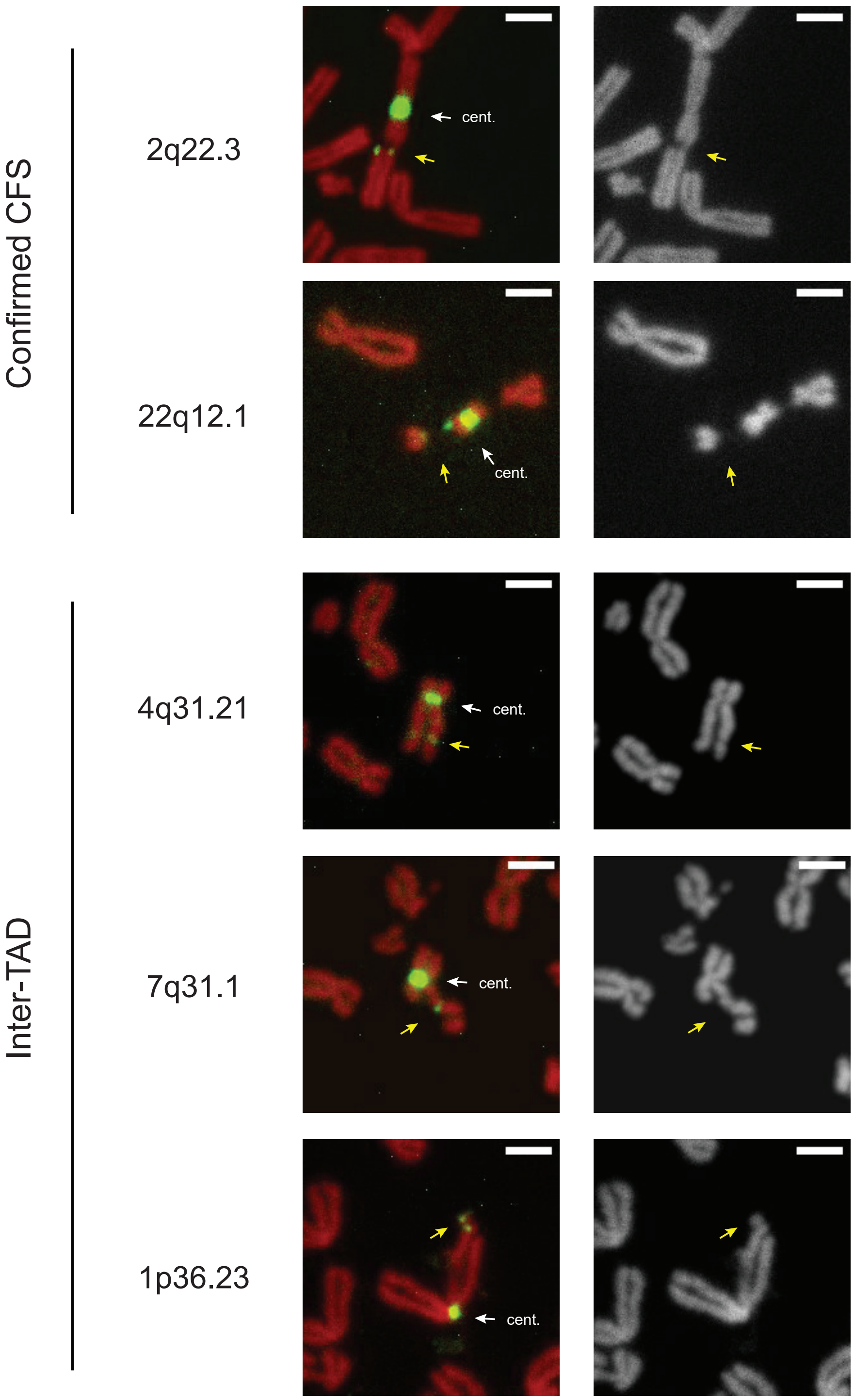

Supplementary Fig. 15. Related to Fig. 4. Chromosomal instability at TAD boundary spanning large expressed genes with RT delay. Molecular FISH mapping identifies recurrent breakage at regions overlapping a TAD boundary with delayed RT and active transcription. Representative images of chromosomal instability at fragility candidate regions overlapping TAD boundaries (inter-TAD) and molecularly confirmed CFSs (Confirmed CFSs) are presented. For quantification of chromosomal instability from two independent experiments please see Fig. 4n and Supplementary Table 5. Scale bars: 2 μm.

Supplementary Figure 16

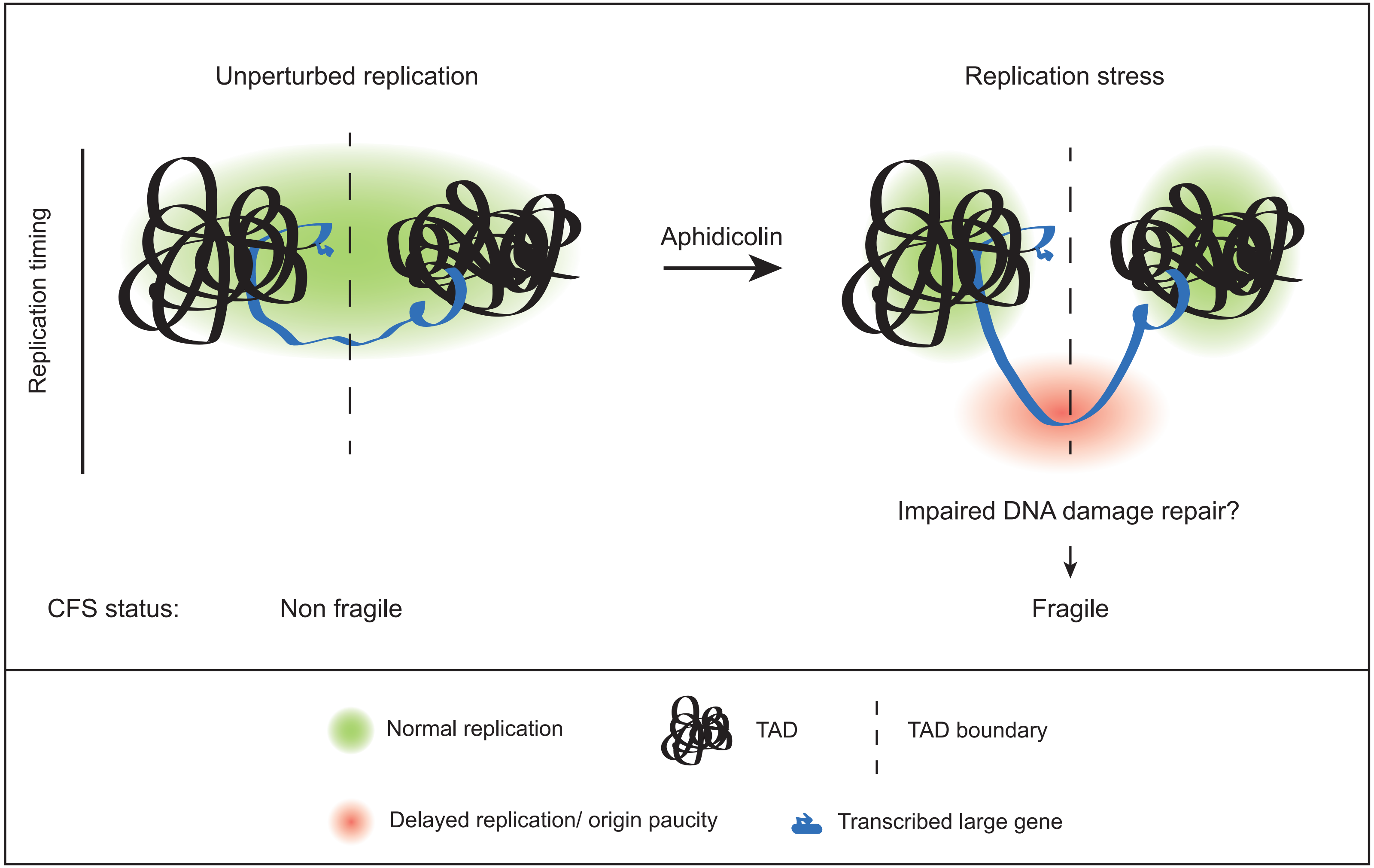

Supplementary Fig. 16. Model of recurrent chromosomal instability under replication stress. Illustration of a fragile site under unperturbed and replication stress conditions. Under unperturbed replication the region is chromosomally stable. However, following replication stress the RT of an origin poor, large expressed gene spanning over a TAD boundary is delayed. The TAD boundary, which may impede proper DNA damage repair, is centered at the core replication sterss sensitive locus of the RT delay and thus promotes chromosomal instability under replication stress.
