# Supplementary material for: 3D genome organization contributes to genome instability at fragile sites
Source: Nat Commun. 2020 Jul 17;11:3613. doi: 10.1038/s41467-020-17448-2 (PMC7367836; doi:10.1038/s41467-020-17448-2)
Supplement: Supplementary file 3 — Description of Additional Supplementary Files [file 41467_2020_17448_MOESM3_ESM.pdf]

Description of Additional Supplementary Files

File Name: Supplementary Data 1
Description: **RT signatures.** List of genomic regions within each RT signature. 1277 windows of 100kb identified as RT variable between Ctrl and APH-treated cells are presented.

File Name: Supplementary Data 2
Description: **Averaged RT profiles of genes.** List of averaged RT profiles for genes clustered into 18 groups according to size. Genes shorter than 5kb were filtered out of the analysis.

File Name: Supplementary Data 3
Description: **Fragility signature candidate genes.** List of large (>300kb), highly transcribed genes with delayed RT upon APH treatment. Whether a gene is inter-TAD or intra-TAD is marked.

File Name: Supplementary Data 4
Description: **Summary of characteristics of large genes (>300kb).** Large genes are divided and counted according to genome architecture (inter- or intra-TAD); transcription (high or low, above or below the median, respectively); RT profile following APH treatment (Delayed or not delayed).

File Name: Supplementary Data 5
Description: **Related to Fig. 4. Summary of chromosomal instability at fragility candidate regions.** Summary of chromosomal instability, breaks and gaps, at regions with fragility signature: Large highly transcribed genes spanning TAD boundaries with delayed RT following APH treatment (inter-TAD), and in control regions of large genes, highly transcribed with delayed RT located within a TAD domain (intra-TAD).
