# Supplementary material for: 3D genome organization contributes to genome instability at fragile sites
Source: Nat Commun. 2020 Jul 17;11:3613. doi: 10.1038/s41467-020-17448-2 (PMC7367836; doi:10.1038/s41467-020-17448-2)
Supplement: Supplementary file 9 — Reporting Summary [file 41467_2020_17448_MOESM9_ESM.pdf]

nature research

Corresponding author(s): David M. Gilbert and Batsheva Kerem

Last updated by author(s): Jun 10, 2020

Reporting Summary

Nature Research wishes to improve the reproducibility of the work that we publish. This form provides structure for consistency and transparency in reporting. For further information on Nature Research policies, see Authors & Referees and the Editorial Policy Checklist.

Statistics

For all statistical analyses, confirm that the following items are present in the figure legend, table legend, main text, or Methods section.

n/a	Confirmed	
☐	☒	The exact sample size (*n*) for each experimental group/condition, given as a discrete number and unit of measurement
☐	☒	A statement on whether measurements were taken from distinct samples or whether the same sample was measured repeatedly
☐	☒	The statistical test(s) used AND whether they are one- or two-sided<br>*Only common tests should be described solely by name; describe more complex techniques in the Methods section.*
☐	☒	A description of all covariates tested
☐	☒	A description of any assumptions or corrections, such as tests of normality and adjustment for multiple comparisons
☐	☒	A full description of the statistical parameters including central tendency (e.g. means) or other basic estimates (e.g. regression coefficient) AND variation (e.g. standard deviation) or associated estimates of uncertainty (e.g. confidence intervals)
☐	☒	For null hypothesis testing, the test statistic (e.g. *F*, *t*, *r*) with confidence intervals, effect sizes, degrees of freedom and *P* value noted<br>*Give P values as exact values whenever suitable.*
☒	☐	For Bayesian analysis, information on the choice of priors and Markov chain Monte Carlo settings
☒	☐	For hierarchical and complex designs, identification of the appropriate level for tests and full reporting of outcomes
☐	☒	Estimates of effect sizes (e.g. Cohen's *d*, Pearson's *r*), indicating how they were calculated

Our web collection on statistics for biologists contains articles on many of the points above.

Software and code

Policy information about availability of computer code

Data collection	The data used in the current study have been either generated for the purpose of the study or downloaded directly from ENCODE and 4DN web portal, no additional software or code was used to collect the data
Data analysis	The Repli-seq, Bru-seq, Hi-C abd CTCF ChIP-seq data were analyzed using R (v3.6.1). The computer codes and further processing data are available on the GitHub repository (https://github.com/dvera/genmat/). Additional codes are available upon request.<br>Fiji/ImageJ (v2.0.0-rc-43) was used for image pre-processing.

For manuscripts utilizing custom algorithms or software that are central to the research but not yet described in published literature, software must be made available to editors/reviewers. We strongly encourage code deposition in a community repository (e.g. GitHub). See the Nature Research guidelines for submitting code & software for further information.

Data

Policy information about availability of data

All manuscripts must include a data availability statement. This statement should provide the following information, where applicable:

- Accession codes, unique identifiers, or web links for publicly available datasets
- A list of figures that have associated raw data
- A description of any restrictions on data availability

All sequencing files and processed count matrices were deposited in Gene Expression Omnibus (GEO) under accession numbers (“GSE150354” [https://www.ncbi.nlm.nih.gov/geo/query/acc.cgi?acc=GSE150354]; and “GSE150543” [https://www.ncbi.nlm.nih.gov/geo/query/acc.cgi?acc=GSE150543]).
Raw Hi-C data of immortalized human foreskin fibroblasts (HFF-hTERT) were downloaded from the 4DN project (“4DNESB6MNCFE” [https://data.4dnucleome.org/experiment-set-replicates/4DNESB6MNCFE/]).
The CTCF ChIP-seq data of HFF cells from the ENCODE project (“GSM1022644” [https://www.ncbi.nlm.nih.gov/geo/query/acc.cgi?acc=GSM1022644]) was used in this work.

Field-specific reporting

Please select the one below that is the best fit for your research. If you are not sure, read the appropriate sections before making your selection.

☒ Life sciences ☐ Behavioural & social sciences ☐ Ecological, evolutionary & environmental sciences

For a reference copy of the document with all sections, see nature.com/documents/nr-reporting-summary-flat.pdf

Life sciences study design

All studies must disclose on these points even when the disclosure is negative.

Sample size	Four biological replicates of Repli-seq experiments of immortalized human foreskin fibroblasts (BJ_hTERT) were performed with and without aphidicolin treatment (0.2uM for 24hr).<br>Two biological replcates of Bru-seq experiments of immortalized human foreskin fibroblasts (BJ_hTERT) were performed with and without aphidicolin treatment (0.2uM for 24hr).<br>Sample size was chosen accordingly to previous publications and in order to overcome technical variation.
Data exclusions	RT profiles of genes were drawn for genes >5 kb in length, since RT data was binned into 5 kb nonoverlapping windows.
Replication	The Repli-seq, Bru-seq and FISH data were repreduciable between biological replicates, from at least two independent experiments.
Randomization	Randomization is not relevant to our study as we aim to compare the replication dynamics and transcription of cells with or without aphidicolin treatment
Blinding	Blinding is not relevant to our study as we aim to compare the replication dynamics and transcription of cells with or without aphidicolin treatment

Reporting for specific materials, systems and methods

We require information from authors about some types of materials, experimental systems and methods used in many studies. Here, indicate whether each material, system or method listed is relevant to your study. If you are not sure if a list item applies to your research, read the appropriate section before selecting a response.

Materials & experimental systems

n/a	Involved in the study
☐	☒ Antibodies
☐	☒ Eukaryotic cell lines
☒	☐ Palaeontology
☒	☐ Animals and other organisms
☒	☐ Human research participants
☒	☐ Clinical data

Methods

n/a	Involved in the study
☒	☐ ChIP-seq
☒	☐ Flow cytometry
☒	☐ MRI-based neuroimaging

Antibodies

Antibodies used	anti-BrdU monoclonal antibody (BD Biosciences, 347580); Stock concentration was diluted to a final concentration of 12.5 μg/ml.<br>goat anti-mouse Alexa Fluor 488 (Invitrogen, A11001);1:200 dilution.
Validation	For anti-BrdU antibody, as described by the manufacture:<br>Anti-BrdU, clone B44, is derived from hybridization of mouse Sp2/0-Ag14 myeloma cells with spleen cells from BALB/c mice immunized with iodouridine-conjugated ovalbumin; Research applications - Cells in G1, S, and G2 + M phases of the cell cycle by flow cytometry; Cell proliferation in the presence of cytotoxic drugs; Sister chromatid exchange using low levels of BrdU.<br><br>For goat anti-mouse Alexa Fluor 488, as described by the manufacture Product Specific Information (https://www.thermofisher.com/antibody/product/Goat-anti-Mouse-IgG-H-L-Cross-Adsorbed-Secondary-Antibody-Polyclonal/A-11001):<br>To minimize cross-reactivity, these goat anti-mouse IgG whole antibodies have been cross-adsorbed against human IgG and human serum. Cross-adsorption or pre-adsorption is a purification step to increase specificity of the antibody resulting in higher sensitivity and less background staining. The secondary antibody solution is passed through a column matrix containing immobilized serum proteins from potentially cross-reactive species. Only the nonspecific-binding secondary antibodies are captured in the column, and the highly specific secondaries flow through. The benefits of this extra step are apparent in multiplexing/multicolor-staining experiments (e.g., flow cytometry) where there is potential cross-reactivity with other primary antibodies or in tissue/cell fluorescent staining experiments where there are may be the presence of endogenous immunoglobulins. For a highly cross-adsorbed secondary antibody equivalent, please see product Cat. No. A11029.

Eukaryotic cell lines

Policy information about cell lines

Cell line source(s)	Immortalized human foreskin fibroblasts (BJ-hTERT), were kindly provided by Prof. Nissim Benvensity, Hebrew University.
Authentication	None
Mycoplasma contamination	The cell cultures were tested and no mycoplasma contamination was found
Commonly misidentified lines (See ICLAC register)	None
